# The Yin and Yang of Cytoreductive SBRT in Oligometastases and Beyond

**DOI:** 10.3389/fonc.2019.00706

**Published:** 2019-08-02

**Authors:** Benjamin E. Onderdonk, Steven J. Chmura

**Affiliations:** Department of Radiation and Cellular Oncology, The University of Chicago, Chicago, IL, United States

**Keywords:** SBRT, radiation, cytoreduction, immunotherapy, 4-1BB, CSF-1R, TGF-beta, oligometastases

## Abstract

**Background:** Oligometastatic disease has emerged as a possibly distinct metastatic phenotype in numerous cancer histologies. With the advancement in treatment modalities including stereotactic body radiation therapy (SBRT), certain patients may derive benefits from local ablative therapy. SBRT alone has already shown to have potential benefits in certain oligometastatic disease types. However, more understanding of the immunologic modulation and microenvironment is needed to guide which patients may benefit from SBRT alone or with combination therapy, if at all.

**Purpose:** The purpose of this review is to offer an update on the emerging data testing SBRT combined with immunotherapy, review the pro-inflammatory and immunosuppressive effects of the tumor microenvironment, discuss novel molecular targets used to augment the immune response, and review potential methods used to decrease toxicity in order to improve the therapeutic ratio.

Stereotactic body radiation therapy (SBRT) has emerged as a prominent and safe modality for metastasis-directed therapy across histologies and appears to result in long-term disease control (1). Initial Phase 2 trials suggest that this long-term disease control translates into a progression-free survival as well as an overall survival benefit further increasing the excitement for definitive phase 3 trials (2, 3). Potential benefits of this approach are hypothesized to include delaying progression in known metastases, preventing further seeding of new metastases, as well as inhibiting progression of micrometastatic foci (4–6). In terms of the effectiveness of the SBRT on local control, increasing the biologically effective dose (or BED) of radiation correlates with the robustness of tumor control (7–10). These techniques have been shown to be effective even when targeting larger metastases (11), with treated metastasis control ranging from 70 to 90% (12). Ongoing phase III trials are investing whether this approach may lead to improve overall survival in a subset of patients with limited metastatic disease (NRG BR002, LU002, SABR-COMET-3, SABR-COMET-10, and SARON). Here, we will focus on the emerging role for SBRT combined with immunotherapy, the modulation of the tumor microenvironment through the utilization of novel molecular targets, and mechanisms used to decrease toxicity during multi-site SBRT.

Beyond radiation alone for oligometastases, there is great interest in enhancing the effects of both radiotherapy and immunotherapy with combined regimens. The goal of combined therapy is to improve both local control of irradiated metastases and un-irradiated responses outside the radiation field (abscopal effect) (13). This immune response may be further affected by the tumor microenvironment. For instance, an immune-excluded environment, which is characterized by a lack of T-cell infiltration, low TH1 cell activity, and reduced cytotoxic T cells, has been shown to predict for worse response to therapy (14).

Described mechanisms for the improvement in immune modulation include but are not limited to increased tumor antigen exposure, improved antigen presentation by dendritic cells, improved T-cell function, re-priming of T-cells, as well as modulation of immunosuppressive cell populations such as T regulatory cells and myeloid derived suppressor cells (15–17). In addition, direct tumor debulking by radiation may also improve systemic immunotherapy outcomes (18, 19). Moreover, ablating multiple areas of disease may also help to overcome PD-L1/CTLA-4 monoclonal antibody therapy resistance (20, 21). Thus, the pre-clinical data suggest that radiation, and in particular SBRT-like doses, may induce a CD8+ T cell mediated anti-tumor response leading to tumor control of the irradiated tumor and potentially to tumor control outside the radiation field.

However, the initial reports of clinical trials combining SBRT with immunotherapy are difficult to interpret with conflicting results. For example, a recent trial (ASCO 2018) in patients with advanced NSCLC treated with 8 Gy x 3 to a single metastatic site followed by Pembrolizumab demonstrated a doubling in overall response rate (ORR) in a randomized setting (22). In contrast, a similar trial design with Progression-Free Survival (PFS) as an endpoint conducted and recently presented in head and neck cancer patients (using 9 Gy x 3, to a single metastasis) failed to demonstrate a signal of efficacy (23). Given the higher doses of radiation in the head and neck trial compared with the NSCLC trial (BED_10_ of 51.3 vs. 43.2, respectively), and the type of immunotherapy were similar (both PD-1 monoclonal antibodies), the difference may lay in the timing of SBRT with immunotherapy. Since the NSCLC trial used sequential SBRT followed by Pembrolizumab and the head and neck trial used SBRT between doses of Nivolumab, the SBRT timing may serve a purpose to “prime” the immune system for optimal effect. However, this efficacy may be tempered by possibly increasing toxicity from a robust response from sequential administration.

As phase 3 trials continue with SBRT alone and many early phase trials continue combining radiation and immunotherapy, a fundamental question remains: which patients, if any, may benefit from SBRT directed at oligometastases? In order to begin to understand this issue, one must incorporate tumor biology into one's treatment paradigm since activation of various cellular pathways may identify potentially curable oligometastatic states (24). Radiation therapy may modulate the tumor microenvironment through pro-immunogenic and immunosuppressive signals (see Figure 1), and the balance of these signals may determine the effectiveness of local tumor cell killing and systemic antitumor immune response. Once we have a better understanding of the immunosuppressive and pro-immunogenic actions of radiation, we can begin to understand which patients may benefit from cytoreductive SBRT alone or in combination with molecular targets. Below we are going examine a few examples of current attempts to modulate the tumor microenvironment to be more favorable toward an SBRT and immunotherapy approach where early stage therapeutics exists for both pre-clinical and clinical testing.

**Figure 1:**
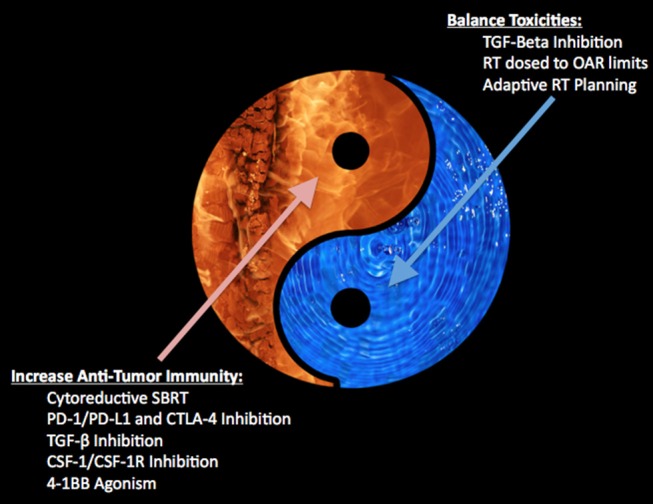
Depicts the balance between increasing anti-tumor immunity with toxicity within the tumor microenvironment.

One method of modulating the microenvironment in favor of SBRT and immunotherapy may be achieved by augmenting pro-inflammatory effects. For example, 4-1BB (CD137) stimulation promotes survival and cell cycle progression of activated human CD8+ T cells while simultaneously inhibiting regulatory T cells (T-regs) (25). Due to these effects, there are numerous ongoing trials using 4-1BB ligands in combination with chemoradiation (NCT00461110), other targeted monocloncal antibody targets (NCT01775631, NCT02110082), and PD-1 inhibitors (NCT03792724, NCT02845323). As these trials examine the combination of 4-1BB ligands with one other modality, our institution is currently evaluating the safety of combining a 4-1BB ligand (Uralumab) with a PD-1 inhibitor (Nivolumab) and SBRT (NCT03431948). These clinical trials will gauge the safety of combination therapy, and may prove useful in identifying future directions for this molecular target.

Another method of improving the therapeutic ratio for SBRT and immunotherapy may come through inhibiting immunosuppressive signals. Activation of these signals result in increased infiltration by T-regs and myeloid-derived suppressor cells (MDSCs). One such pathway with a molecular target includes the macrophage colony-stimulating factor 1 receptor pathway (CSF-1R). CSF-1R signaling has specifically demonstrated a role in differentiation, maintenance, trafficking, functioning of the monocytic lineage, and serves as a prominent driver in resident tumor macrophages (26, 27). These tumor-associated macrophages (TAMs) have shown the ability to promote tumor regrowth (28). Although the relationship between TAMs is relatively plastic, the ratio of M1/M2 TAMs is prognostic of clinical outcome in multiple of human cancers including lung, breast, pancreas, and lymphoma (29, 30). There is evidence of a benefit in cancer therapies by targeting these trophic effects of TAMs through this CSF-1R pathway (31, 32). Moreover, further research targeting this receptor in combination with other therapies (i.e., PD-1/PD-L1 inhibitors) are currently underway. For example, preliminary phase 1a/1b data (SITC 2018) in pancreatic cancer demonstrated safety of Cabiralizumab (CSF-1R inhibitor) with Nivolumab (PD-1 inhibitor) with a 6-month disease control rate of 13 percent and an ORR of 10 percent (33). Given this preclinical and early clinical data, our institution is currently investigating the toxicity of SBRT, PD-1 inhibition (Nivolumab) with a CSF-1R monoclonal antibody (Cabiralizumab (NCT03431948) in a phase 1 setting. Further trials examining this molecular target may identify subsets of patients that may benefit from combination of SBRT and immunotherapy.

Beyond modulating the immune microenvironment through 4-1BB agonism or CSF-1R inhibition, TGF-β in particular has shown to be a primary mechanism of tumor immune evasion by blocking the TH-1 effector phenotype, inhibit T cell division/function and natural killer (NK) cell function, and by promoting epithelial-mesenchymal transition (EMT) (34, 35). This microenvironment high in TGF-β signaling has further characterized a poor-prognostic phenotype in colorectal cancer, and preclinical models showed that inhibition of TGF-β stops disease progression in liver metastases from colon cancer (36). Moreover, a lack of response to PD-L1 monotherapy has been associated with increased TGF-β by creating an immune-excluded phenotype, and several preclinical studies examining the utility of TGF-β inhibition in promoting PD-L1 response (34, 37). In addition to these immune-exclusion aspects of TGF-β, increased TGF-β signaling has demonstrated radiation resistance (38), while inhibition of TGF-β cell lines promoted radiation sensitization (22). Thus, targeting these immune-excluding signaling molecules, may allow for improved response to PD-1/PD-L1 therapy and radiation therapy.

Another method of overcoming local PD-1/PD-L1 immune resistance is through the use of novel bispecific fusion protein technology. One such fusion protein, (M7824), combines an anti-PD-L1 monoclonal antibody with two TGF-β receptor 2 molecules to serve as a TGF-β “Trap.” Urothelial cancer cell lines treated with this bispecific fusion protein demonstrated an increase in T-cell trafficking, TRAIL-mediated tumor lysis, and ADCC when compared to PD-L1 inhibition alone (39). In NSCLC, this bifunctional PD-L1 and TGF-β inhibitor prevented tumor endogenous mesenchalization compared with PD-L1 inhibition alone and enhance antibody-dependent cellular cytotoxicity (ADCC) in cervical, breast, and prostate cancer cell lines (40, 41). Also, when compared to PD-L1 monotherapy alone, this bifunctional inhibition demonstrated a decrease in TAMs and increases effector T cells, M1 cells, and improved the M1/M2 ratio (discussed above) (35). Combining this bispecific fusion protein with radiation therapy may promote an immune response while simultaneously decreasing immune exclusion. In pre-clinical models, this bifunctional inhibitor combined with radiation therapy promoted the inhibition of tumor growth, metastases and improved survival (42). Furthermore, murine models combining radiotherapy and a the bifunctional protein results in significantly greater irradiated tumor response as well as a secondary non-irradiated tumor response, suggesting secondary systemic beneficial effects of radiation on the immune response (35). With this new preclinical and early clinical data in mind, these novel bispecific monoclonal antibodies may prove beneficial in subsets of patients with oligometastatic disease and further testing is warranted.

As radiation therapy may promote pro-inflammatory effects in the tumor microenvironment, it may also promote immune-exclusion. For example, a preclinical study demonstrated that high doses of radiation has shown the ability to induce an immune-suppressive, M2-like phenotype, and that reversing this effect could improve local control of tumors and stimulate a more robust immune response (43). Thus, some patients receiving cytoreductive SBRT may gain a benefit from concomitant use of targetable signaling molecules (PD-1/PD-L1, 4-1BB, CSF-1R, and TGF- β) in order to limit the immune-exclusion and promote a more robust response to SBRT.

Beyond the initial concept of oligometastatic disease, the largest published benefit of immunotherapy to date comes from the PACIFIC Trial. Although these patients all had locally advanced non-small cell lung, presumably they may have had micrometastatic disease. In those treated with adjuvant Durvalumab, there was a tripling of median PFS and a 10% improvement in 2-year OS (44, 45). Thus, we hypothesize that patients with the lowest burden of metastatic disease, in this case possibly micrometastatic disease, may benefit the most from immunotherapy. Although a radiation-immunotherapy interaction may also be the rationale for this benefit, further surgical studies in locally advanced NSCLC combined with immunotherapy (i.e., ECOG-ACRIN E5142) may aid the argument of minimal micrometastatic disease benefitting the most from immunotherapy. On the opposite end of the disease spectrum, a recent prospective phase 1 trial from our group further supports the notion of possibly improving on the immune-exclusion microenvironment through cytoreduction of metastatic disease with high-dose SBRT and combined with pembrolizumab. The combination demonstrated a long median overall survival (9.6 months) with low rates of severe toxicity in a heavily pre-treated, non-oligometastatic patient cohort (46). Moreover, local control was no different between partial tumor radiation coverage (see Figure 2) for larger tumors and smaller ones suggesting a synergistic effect with the immunotherapy. In an exploratory analysis, the only predictor for overall survival was the local control of the irradiated tumor with high dose radiation (47). As oligometastatic states exist between “micrometastatic” and heavily pre-treated metastatic states, evidence from our group further confirms the necessity for local control of large metastatic lesions through cytoreductive SBRT to promote an inflammatory microenvironment and limit immune-exclusion.

**Figure 2:**
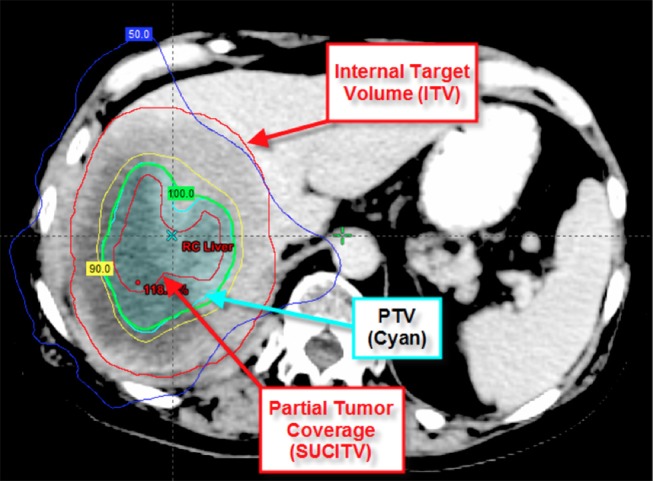
Depicts partial tumor coverage of a large liver lesion. The 45 Gy in three fractions was prescribed to the central 65 cc of the tumor (SUCITV) with a 5 mm PTV expansion. ITV, internal target volume; SUCITV, seriously undercovered immuno-target volume; PTV, planning target volume.

SBRT alone to oligometastases is currently well-tolerated with around 30% grade 2+ toxicity, however given the grade 5 toxicities observed, there is continued concern that toxicity may increase with multi-site SBRT (3). These pro-immunologic benefits of SBRT, and especially with the combination with other pro-inflammatory molecular targets, may result in improved outcomes however there is concern over increased toxicity. Some strategies to limit toxicity are attempting to ameliorate this inflammatory response in the normal tissues (see Figure 1). For example, a recent study demonstrated that patients with non-small cell lung cancer showed an increased risk for radiation pneumonitis with increased TGF-β signaling (48), while a preclinical study demonstrated a decrease risk for radiation pneumonitis in mice treated with TGF-β inhibition (49). Thus, inhibition of TGF-β, possibly in the bispecific antibody approach, may provide another means of limiting the toxicity from therapy. Another possible mechanism of limiting toxicity adopted by our group has been through decreasing dose to adjacent OARs. In patients receiving immunomodulatory agents, initial observation suggests that local control may be achieved despite significantly decreased doses to the tumor edge (see Figure 2) (46, 47). With this approach, we noticed that the majority of the center of the tumors received a high dose of radiation while the periphery of the tumor received a lower prescription dose. Interestingly, we were able to increase the dose to the center of the lesion without compromising control, while also limiting our dose to critical structures and subsequent toxicity. Another method of limiting toxicity, although logistically challenging, may be through adaptive radiation planning. This method of re-planning the SBRT treatment during the course of radiation may allow for the most accurate method of delivery as it accounts for tumor responses and relationship of adjacent OARs to target lesions. By using these various methods: TGF-β inhibition, limiting target coverage to spare adjacent OARs, and adaptive radiation planning, we may continue to treat more lesions with multi-site cytoreductive SBRT while also limiting toxicity and not compromising our primary objective of local control.

In conclusion, phase 3 trials of SBRT alone for oligometastasis are currently underway in various histologic disease sites. These studies are needed to demonstrate oligometastatic states while also being cognizant of emerging concepts regarding tumor biology and the microenvironment. As this data emerges, future trials examining cytoreductive SBRT combined with checkpoint inhibition and using novel agents against other molecular targets, such as TGF-β, CSF-1, and 4-1BB may demonstrate a logical progression of the current data. We believe there is promise in further translational and clinical studies identifying a synergistic mechanism to increase anti-tumor immunity through high-dose SBRT combined with systemic therapies. We believe the most promising to date revolve around cytoreduction of tumor burden combined with systemic treatments aimed at balancing toxicities (i.e., TGF-β), partial-tumor coverage of the prescription dose to avoid critical organs, and adaptive radiation planning.

## Author Contributions

BO conducted the literature search and wrote the majority of this manuscript. SC provided academic support and also contributed in writing this manuscript.

### Conflict of Interest Statement

SC declares Consultancy Reflexion, Abviee, Spouse (Astellas). BO declares that the research was conducted in the absence of any commercial or financial relationships that could be construed as a potential conflict of interest. The handling editor declared a past co-authorship with one of the authors SC.
